# Intraarticular injection of liposomal adenosine reduces cartilage damage in established murine and rat models of osteoarthritis

**DOI:** 10.1038/s41598-020-68302-w

**Published:** 2020-08-10

**Authors:** Carmen Corciulo, Cristina M. Castro, Thomas Coughlin, Samson Jacob, Zhu Li, David Fenyö, Daniel B. Rifkin, Oran D. Kennedy, Bruce Neil Cronstein

**Affiliations:** 1 Division of Translational Medicine, Department of Medicine, NYU School of Medicine, 550 First Avenue, New York, NY 10016 USA; 2 Department of Orthopedic Surgery, NYU School of Medicine, 550 First Avenue, New York, NY 10016 USA; 3 Institute for Systems Genetics, NYU School of Medicine, 435 East 30th Street, New York, NY 10016 USA; 4 Department of Biochemistry and Molecular Pharmacology, NYU School of Medicine, 435 East 30th Street, New York, NY 10016 USA; 5 Department of Cell Biology, NYU School of Medicine, 550 First Avenue, New York, NY 10016 USA; 6 Department of Anatomy, The Royal College of Surgeons in Ireland, 123 St Stephens Green, Dublin 2, Ireland; 7 Division of Rheumatology, Department of Medicine, NYU School of Medicine, 550 First Avenue, New York, NY 10016 USA; 8 Medical Science Building, NYU Langone Health, 550 1st Avenue, New York, NY 10016 USA

**Keywords:** Osteoarthritis, Experimental models of disease

## Abstract

Osteoarthritis (OA) affects nearly 10% of the population of the United States and other industrialized countries and, at present, short of surgical joint replacement, there is no therapy available that can reverse the progression of the disease. Adenosine, acting at its A2A receptor (A2AR), is a critical autocrine factor for maintenance of cartilage homeostasis and here we report that injection of liposomal suspensions of either adenosine or a selective A2AR agonist, CGS21680, significantly reduced OA cartilage damage in a murine model of obesity-induced OA. The same treatment also improved swelling and preserved cartilage in the affected knees in a rat model of established post-traumatic OA (PTOA). Differential expression analysis of mRNA from chondrocytes harvested from knees of rats with PTOA treated with liposomal A2AR agonist revealed downregulation of genes associated with matrix degradation and upregulation of genes associated with cell proliferation as compared to liposomes alone. Studies in vitro and in affected joints demonstrated that A2AR ligation increased the nuclear P-SMAD2/3/P-SMAD1/5/8 ratio, a change associated with repression of terminal chondrocyte differentiation. These results strongly suggest that targeting the A2AR is an effective approach to treat OA.

## Introduction

Osteoarthritis (OA) is a common disease affecting 151 million people worldwide and its incidence is expected to increase in industrialized countries due to aging and increased obesity of the population, a condition that together with previous joint injury represent the most common risk factors^1^. OA can affect any joint, but most commonly affects the knee, hip and hand. The prevalence of OA is greatest in the knee joint, in both women (47%) and men (40%), and there is no therapy currently available that can reverse or halt the progression of OA^2,3^ short of total joint replacement. Total knee replacements are the most common joint surgeries and it has been estimated that there will be a fivefold increase in the number of patients undergoing this surgical procedure up to 3.5 million by 2030^4,5^.

In search of effective therapies, a number of different approaches have been taken including a focus on both growth factors, such as transforming growth factor-beta (TGFβ), and other molecular pathways involved in regulating cartilage development and homeostasis. TGFβ molecular signaling exerts dual and opposing roles in cartilage and chondrocyte health depending on the receptor and signal activated downstream. It has been shown that inhibition of high levels of systemic TGFβ attenuates anterior cruciate ligament rupture-induced OA in mice by preventing loss of proteoglycan from the cartilage and protecting the subchondral bone from structural alteration^6^. Moreover, the effect of TGFβ on joint health depends on which receptor it binds. Activation of downstream signaling pathways after TGFβ stimulation of ALK5, which is expressed on chondrocytes, leads to SMAD2/3 phosphorylation and signaling, phenomena associated with maintenance of cartilage. Indeed mice lacking SMAD3 show loss of proteoglycan, collagen 2, aggrecan and express more MMP13 and MMP9^7^. In contrast, signaling through ALK1, also expressed on chondrocytes, leads to SMAD1/5/8 phosphorylation and signaling resulting in chondrocyte hypertrophy^8^. The importance of appropriate TGFβ signaling is more evident in the cartilage of both elderly and osteoarthritis patients who show a decrease of TGFβ2, TGFβ3, and ALK5 expression with increased SMAD1/5/8 translocation to the nucleus^9,10^.

We have recently reported that adenosine, generated extracellularly from the hydrolysis of ATP, is an autocrine homeostatic factor that maintains chondrocyte and cartilage balance^11^. We further demonstrated that intraarticular injection of liposomal preparations of adenosine, prevents cartilage destruction and chondrocyte hypertrophy via interaction with its A2A receptor (A2AR) in a rat model of post-traumatic OA^11^. Published evidence suggests that endogenous adenosine, acting at the A2AR, protects cartilage from degradation and prevents release of degenerative and pro-inflammatory molecules from articular chondrocytes^12-14^. In the clinic, unlike experimental models, treatment for OA begins well after the process has begun and OA has been established. Therefore, we determined in this study whether treatment of established OA with either liposomal preparations of adenosine (Lipo-ADE) or liposomal preparations of CGS21680, a selective A2AR agonist (Lipo-CGS), could prevent progression of OA. Moreover, we explored the mechanism by which A2AR receptor stimulation alters cartilage pathophysiology.

Here we report that intra-articular injections of liposomal preparations of adenosine and the selective A2AR agonist, CGS21680, prevent OA progression in two different models of OA, obesity-induced OA in mice and post-traumatic OA in rats.

## Results

### Intra-articular injection of Lipo-Ade and Lipo-CGS reversed OA in a murine model of obesity-induced OA

**Figure 1:**
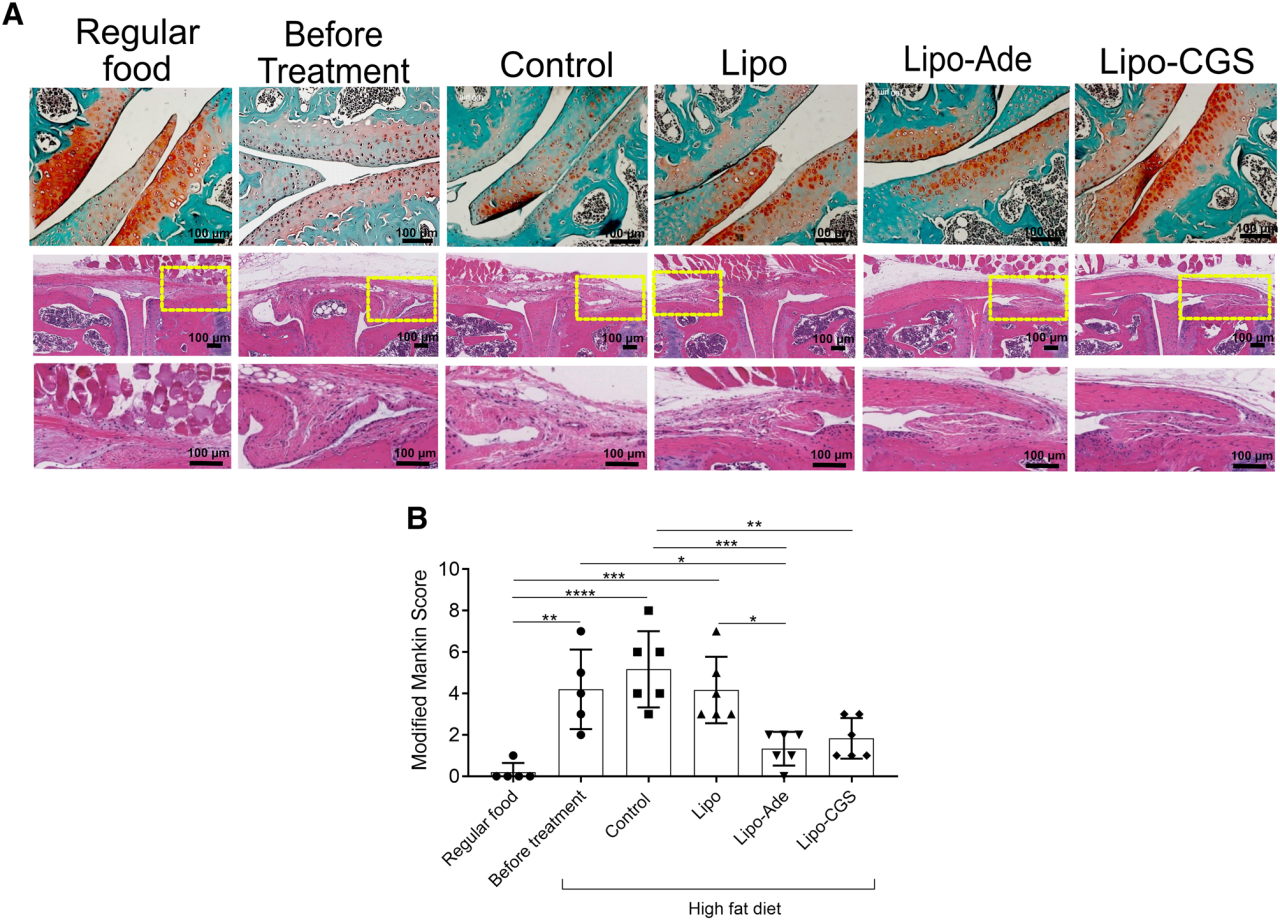
A2AR stimulation blocks OA progression in a murine model of obesity related OA. A. The top section of the figure shows representative images of Safranin- O stained cartilage; on the bottom are representative images of Hematoxylin & Eosin staining of the synovium showing reduction in synovial thickness and infiltration of immune cells in mice treated with Lipo-ADE and Lipo-CGS (the yellow square define the magnified area). Figures labeled “Before Treatment” are of mouse knees collected following 12 weeks of a high fat diet when intraarticular injections would have commenced in the various treatment groups. B. Plotted are the Modified Mankin Scores of the different experimental groups. Data are expressed as mean ± s.e.m. of 6 animals for each group and data were analyzed for statistical significance by one-way analysis of variance followed by Bonferroni post hoc test of differences among various treatments (*p < 0.05; **p < 0.01; ***p < 0.001; ****p < 0.0001).

Obesity is a common risk factor for OA, so we tested the effect of Lipo-Ade and Lipo-CGS in a murine model of obesity-induced OA. Mice were maintained on a high fat (60%) diet; a DEXA scan was performed on all of the mice before and after the high fat diet was started and significant differences in body fat composition and percentage body fat were observed in all the obese animals compared to the group fed with regular chow (Supplemental Fig. 2, *p* < 0.001); no difference was detected among the various treatment groups studied here. In addition, there were no differences in bone mineral density, bone mineral content and lean tissue among all the experimental groups (Supplemental Fig. 2). Immunoassay on plasma samples revealed a significant increase of the pro-inflammatory cytokines IL-6, leptin and resistin in HFD mice compare to the NC fed mice (Supplemental Fig. 3) and TNF-α was undetectable in all of the mice. Treatment of mice with intraarticular injections of Lipo or Lipo-CGS did not affect the levels of these adipokines. In this model of OA treatment with intra-articular Lipo-Ade and Lipo-CGS led to significant histologic improvement in obesity-related OA with restoration of Safranin O-stained proteoglycan in the treated mice, as compared to the saline- or Lipo-treated mice (Fig. 1A). Interestingly, H&E staining showed a reduction in synovial membrane thickness and inflammation in Lipo-CGS- and Lipo-Ade-treated mice as compared to the control groups (Fig. 1A). Knees of mice fed with a high fat diet (HFD mice) had an OARSI score of 5.2 ± 1.8. Injections of both Lipo-Ade and Lipo-CGS dramatically decreased OA severity (OARSI score 1.3 ± 0.8 and 1.8 ± 1.0, respectively, *p* < 0.001 *vs* untreated for both, Fig. 1B).

### Intra-articular injections of either liposomal adenosine (Lipo-Ade) or A2AR agonist (Lipo-CGS) promote cartilage formation in established PTOA

**Figure 2:**
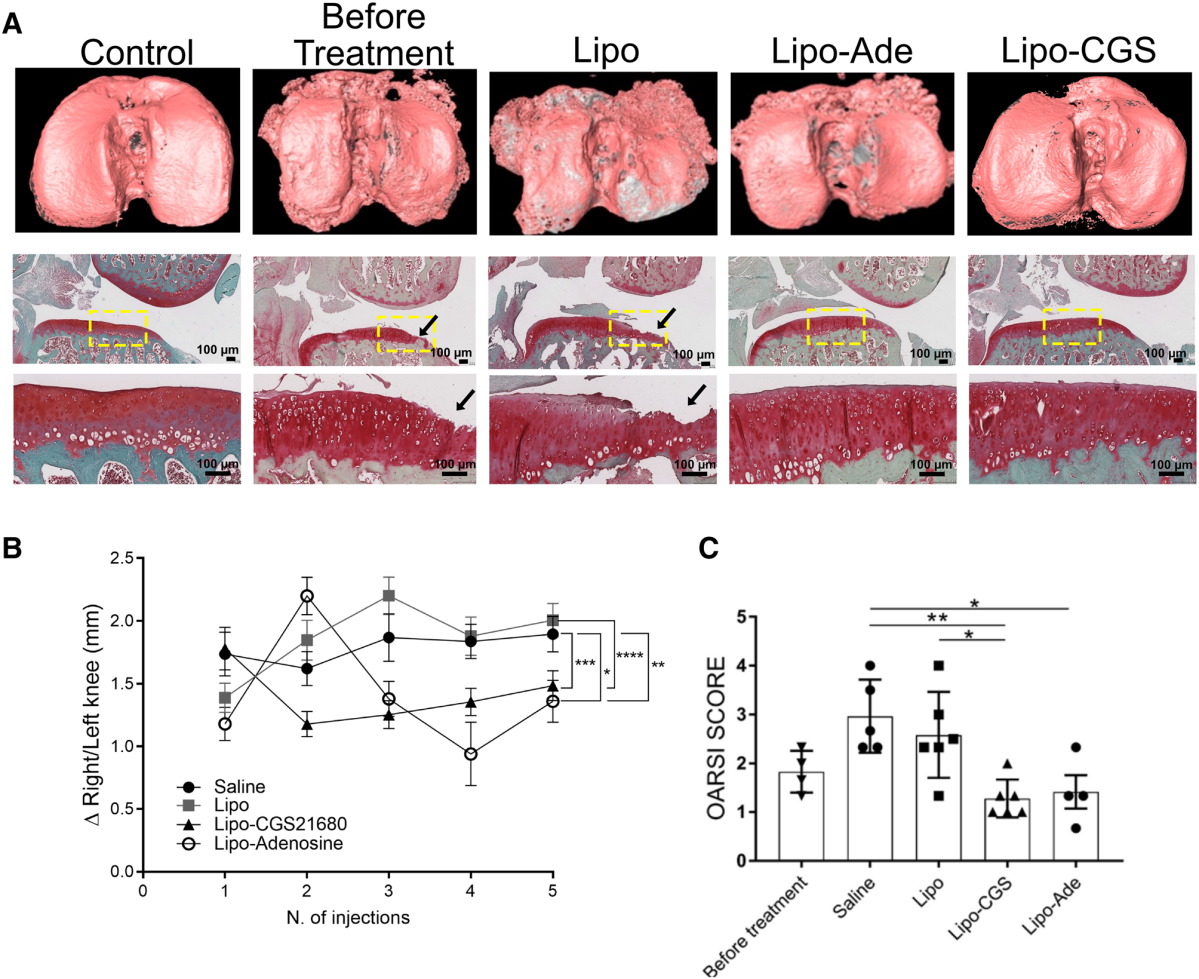
Adenosine and A2AR stimulation promote cartilage formation in an established PTOA model. A. On the top raw representative mCT images of hexabrix-imaged tibia. The cartilage is pink in this image and underlying bone is dark gray. In the row beneath are representative safranin-O-stained sections (the yellow square define the magnified area on the raw below; black arrow are pointing at the region of cartilage damage). Figures labeled “Before Treatment” are of rat knees collected 4 weeks after disruption of ACL when intraarticular injections would have commenced in the various treatment groups. B. Swelling measurement of the knee joint before every injection compare to the healthy knee of each rat for the different experimental group. C. Graphs show the OARSI scores of the knees of the rats studied here. Data are expressed as mean ± S.E.M. of 4–6 animals for each group and data were analyzed for statistical significance by one-way analysis of variance followed by Bonferroni post hoc test of differences among various treatments (*p < 0.05; **p < 0.01; ***p < 0.001; ****p < 0.0001).

Patients with OA generally seek medical care after the onset of OA, and, to date, there are no therapies available that either prevent progression of OA or reverse the loss of the cartilage in OA joints. Therefore, we tested the capacity of Lipo-CGS and Lipo-Ade to improve OA-related changes in cartilage and chondrocytes in the rat model of PTOA in which the first injection was performed 4 weeks after the ACL injury, at a time when OA is well established^15^. MicroCT reconstruction of the affected tibias demonstrated clear changes in bone morphology and loss of cartilage in PTOA knees examined 4 weeks after injury, at a time point prior to the start of intra-articular injections, consistent with the loss of Safranin O staining and diminished cartilage thickness (Fig. 2A). At the end of the experimental treatment, there was a more dramatic loss of cartilage and bony sclerosis observed in the Lipo-treated knees (Fig. 2A). In contrast, while there was still apparent injury of the bone in the Lipo-CGS21680- and Lipo-Ade treated rats, there appeared to be reduced loss of cartilage by both microCT and histology (Fig. 2A). Intra-articular injection of Lipo-CGS markedly reduced knee swelling, as compared to the rats injected with Lipo or saline (Fig. 2B). In contrast, intraarticular injection of Lipo-Ade led to an initial increase in swelling after the first injection which was markedly reduced 10 days later at the time of the next injection. The apparent increase in knee swelling induced by Lipo-Ade injections may be due to non-selective stimulation of A2B receptors which have previously been shown to exacerbate swelling of joints in the adjuvant arthritis model in rats^16^. Quantitative histologic examination (OARSI Score) showed significant OA changes in the knees of rats 4 weeks after ACL rupture and modest progression of the OA in the saline and Lipo-treated knees and progression was completely prevented in the Lipo-CGS and Lipo-Ade injected knees (Fig. 2C). The articular cartilage in the saline- and lipo-treated knees is eroded and, in some areas, underlying bone is showing through (Fig. 2A) but, surprisingly, the cartilage appearance remained stable in the knees of rats treated with either intra-articular saline or empty liposomes.

The cartilage in the treated knees is not fibrocartilage, since there is little col1a1 expressed in the treated cartilage (Supplemental Fig. 4). Moreover, col2a1 is more highly expressed in Lipo-Ade- and in Lipo-CGS-treated rats compared to the Saline- and Lipo-treated group (Supplemental Fig. 4). In summary, the treatment of rats with PTOA with either Lipo-CGS or Lipo-Ade prevented progression of OA and increased the volume of articular cartilage in established PTOA.

### A2AR agonist regulates genome-wide gene expression in established PTOA rat model

**Figure 3:**
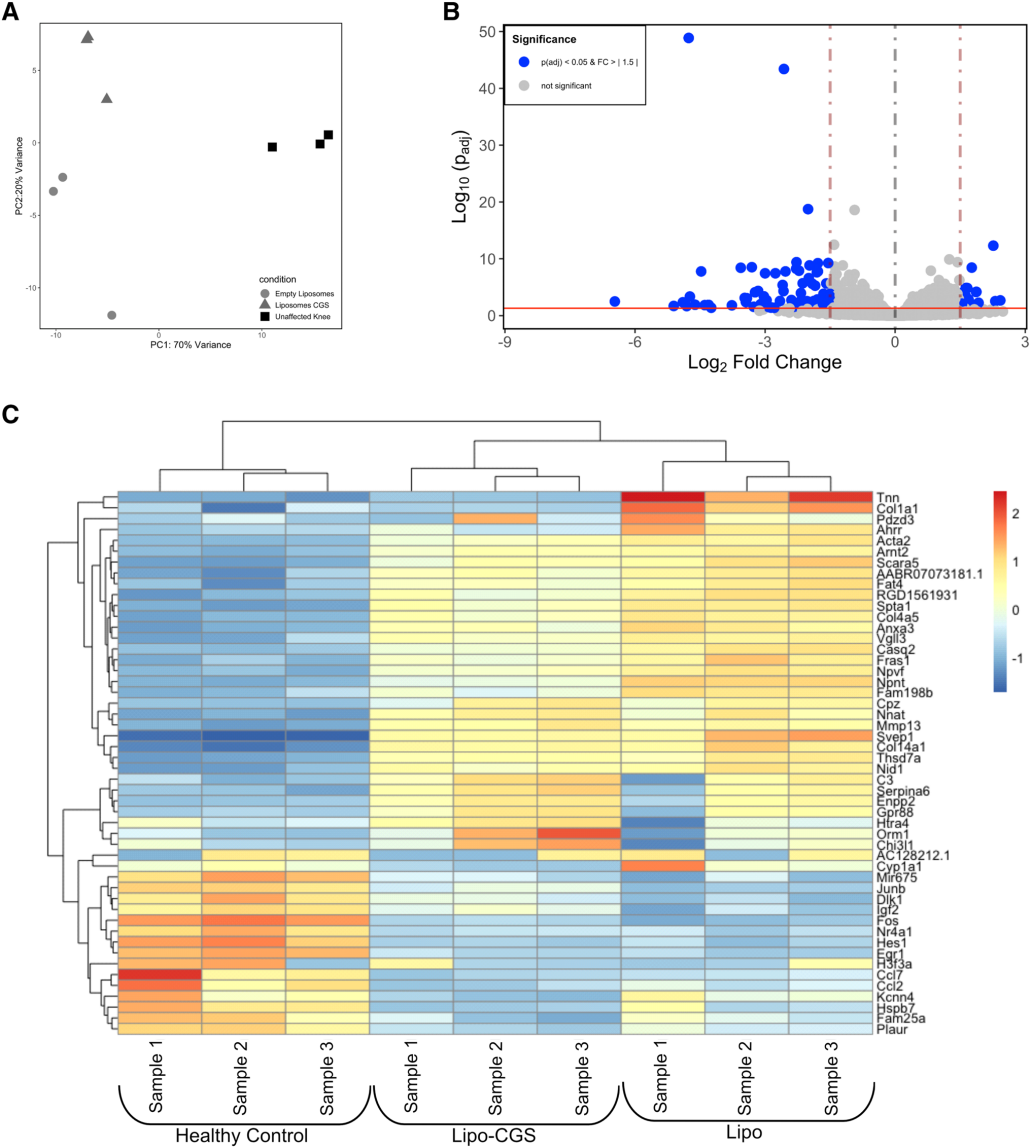
Transcriptome alteration in knee chondrocytes after Lipo-CGS21680 intraarticular treatment. Transcriptome analysis of articular chondrocytes isolated from PTOA rats. (**A**) Principal component analysis of RNA-seq expression data demonstrates distinct separation between healthy (unaffected knee), vehicle (empty liposomes) and Lipo-CGS21680-treated (Liposomes_CGS) groups. (**B**) 8 genes were upregulated and 42 were down-regulated in Lipo-CGS21680 group compare to the vehicle treated group (volcano plot). (**C**) Heatmap showing the first 50 genes alterated by Lipo or Lipo-CGS treatment.

**Figure 4:**
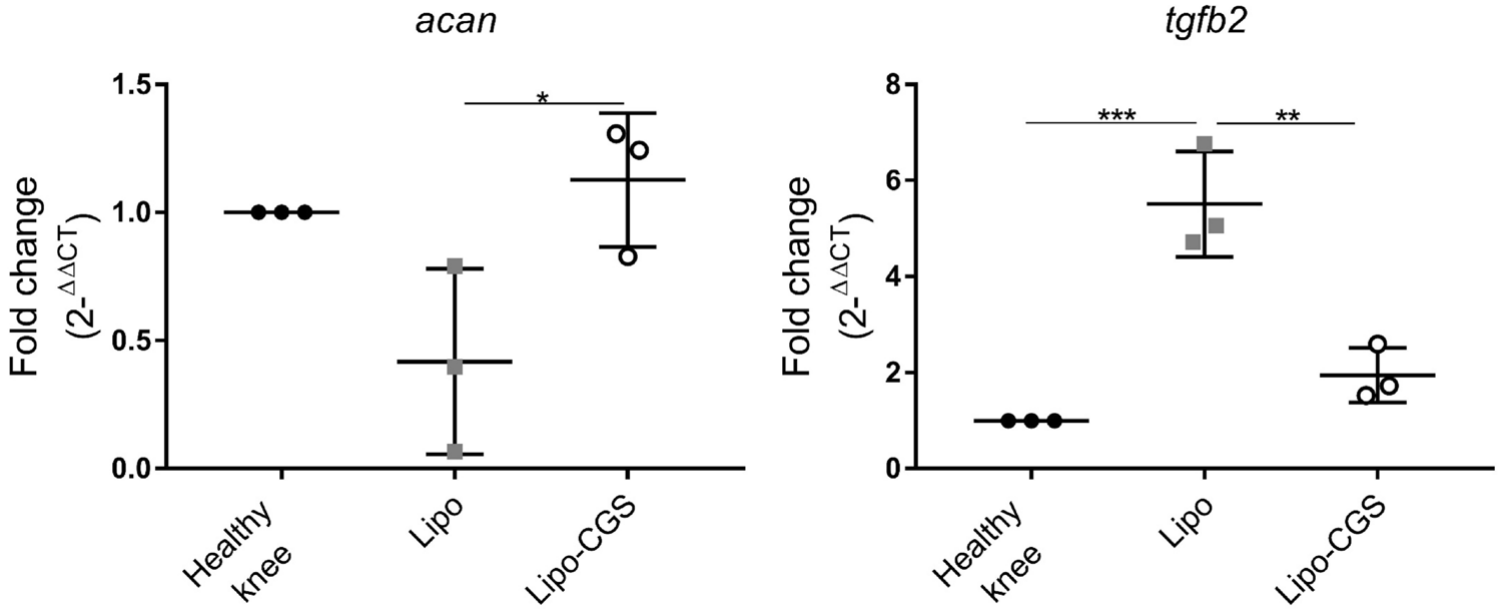
Transcriptome alteration in knee chondrocytes after Lipo-CGS21680 intraarticular treatment. RT-PCR results for aggrecan and TGF-β2 genes in articular chondrocytes isolated from PTOA rats (n = 3 for each group; *p < 0.05; **p < 0.01; ***p < 0.001).

To better understand how A2AR stimulation reverses OA, we carried out genome wide expression analysis in chondrocytes isolated from knees of rats with PTOA treated with Lipo or Lipo-CGS or from normal knees. Principal component analysis of the resulting changes in mRNA shows clear separation among the normal, Lipo or Lipo-CGS-treated chondrocytes (Fig. 3A). We next identified the genes that were differentially regulated between Lipo and Lipo-CGS-treated (> 1.5 fold change, *p* < 0.0005) and plotted them in Fig. 3B and C. Pathway analysis of these results reveals that Lipo-CGS regulated a number of pathways (Supplemental Fig. 5). Consistent with the changes observed histologically and by microCT, we found that there was upregulation of cartilage anabolic genes and downregulation of genes associated with chondrocyte hypertrophy, cartilage catabolism and apoptosis (Fig. 4 and Table 1). We have previously confirmed the changes in many of the genes in either published work or by RT-PCR and further confirmed these changes here (Table 1). Interestingly, there also appeared to be a decrease in TGFβ-3 expression in the Lipo-CGS-treated chondrocytes as compared to the Lipo-treated chondrocytes (Fig. 3C).

**Table 1:** Gene pathways modulated in knee chondrocytes after Lipo-CGS21680 intraarticular treatment.

Pathway	Gene name	Lipo-CGS versus Lipo
Chondrocyte hypertrophy	Col1a1*	↓ *p* < 0.001
	*Col3a1*	↓ *p* < 0.001
	*Col4a5*	↓ *p* < 0.01
Cartilage catabolism	Adamts14*	↓ *p* < 0.001
	*Adamts9*	↓ *p* < 0.001
	*Mmp23*	↓ *p* < 0.05
	*Mmp28*	↓ *p* < 0.001
Apoptosis	Ahhr*	↓ *p* < 0.001
	*S1PR3*	↓ *p* < 0.001
TGF-β	Tgfb3*	↓s *p* < 0.001

*Changes in these messages were validated by RT-PCR. All *p* values listed are adjusted for multiple comparisons.

**Figure 5:**
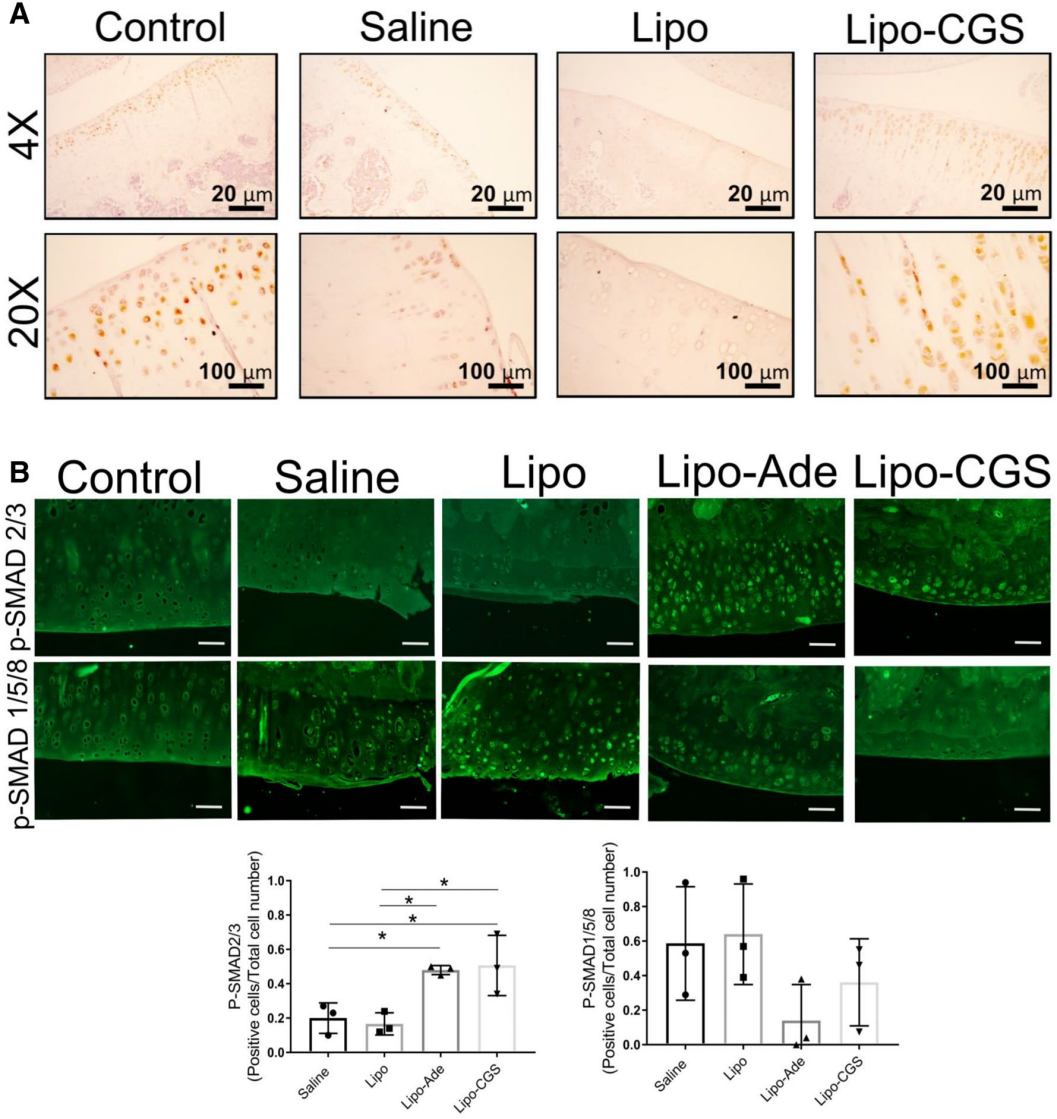

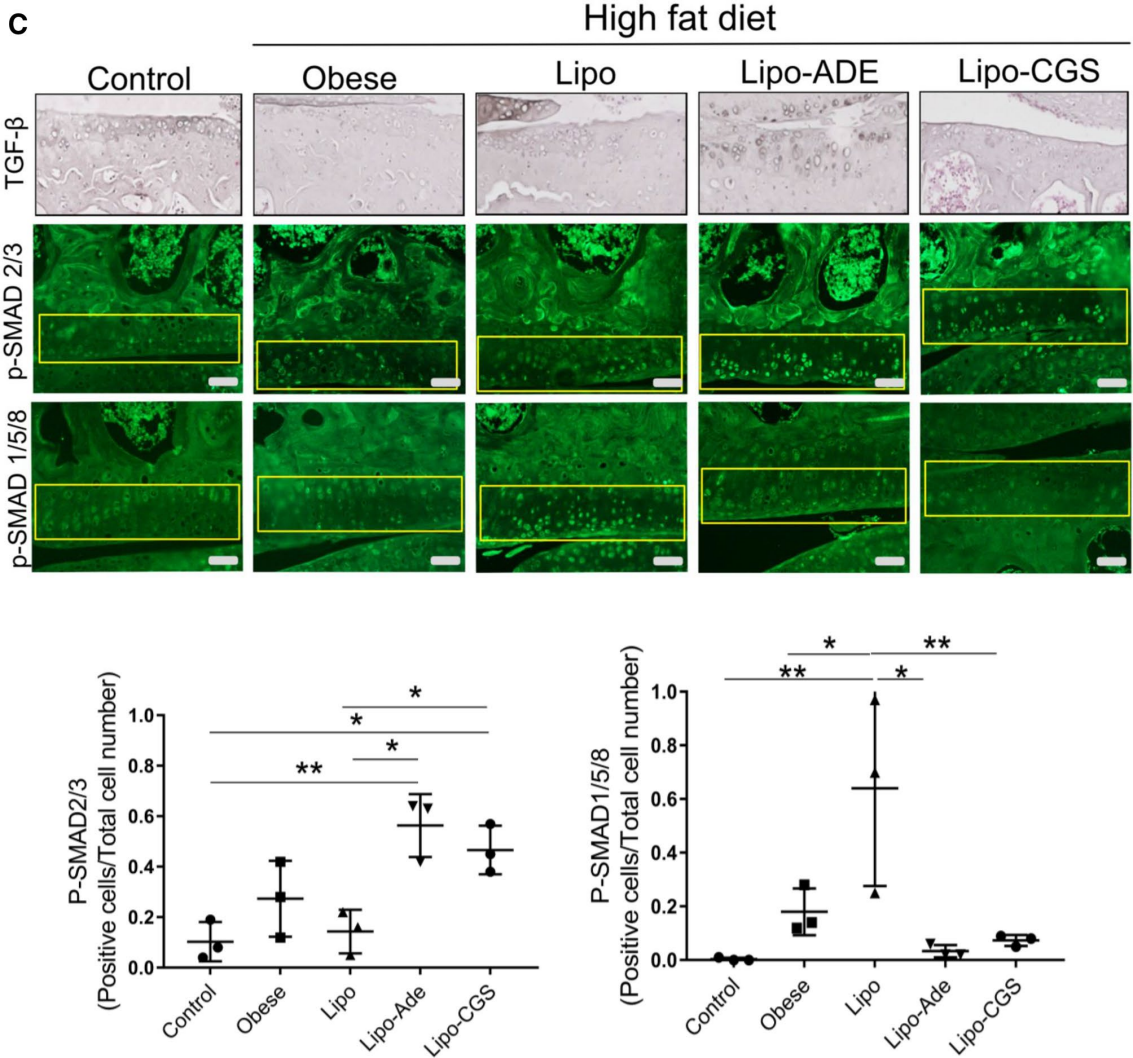
A2AR activation induces switch in TGF-β molecular signaling from phosphorylation of SMAD2/3 to SMAD1/5/8 pathway. (**A**) Representative immunohistochemistry pictures of TGF-β expression in OA rats articular cartilage after different treatments. (**B**) P-SMAD2/3 activation in chondrocytes of rat knees treated with Lipo-CGS. The original magnification of these figures was 40× (the yellow square defines the area of articular cartilage). Plotted below are the number of SMAD1/5/8- and SMAD2/3-positive cells in the cartilage of knees treated with saline, empty liposomes or lipo-ade or lipo-CGS. Each point represents the ratio of positive/total cells from a section from a different animal. Statistical significance was determined by 1-way ANOVA with repeated measures followed by Bonferroni’s post hoc test for differences between groups; (**C**) Shift of SMAD activation from P-SMAD2/3 to P-SMAD1/5/8 in Lipo-CGS and Lipo-ADE treated OA knee in murine model.

We further confirmed these findings in vitro using neonatal murine chondrocytes and demonstrated that the A2AR agonist CGS21680 stimulated increased chondrocyte aggrecan production, an effect reversed by the A2AR-selective antagonist ZM241385 (Supplemental Fig. 6A). Moreover, CGS21680 stimulated an increase in message for col2a1 which was exaggerated after IL-1 treatment of the chondrocytes (Supplemental Fig. 6B).

**Figure 6:**
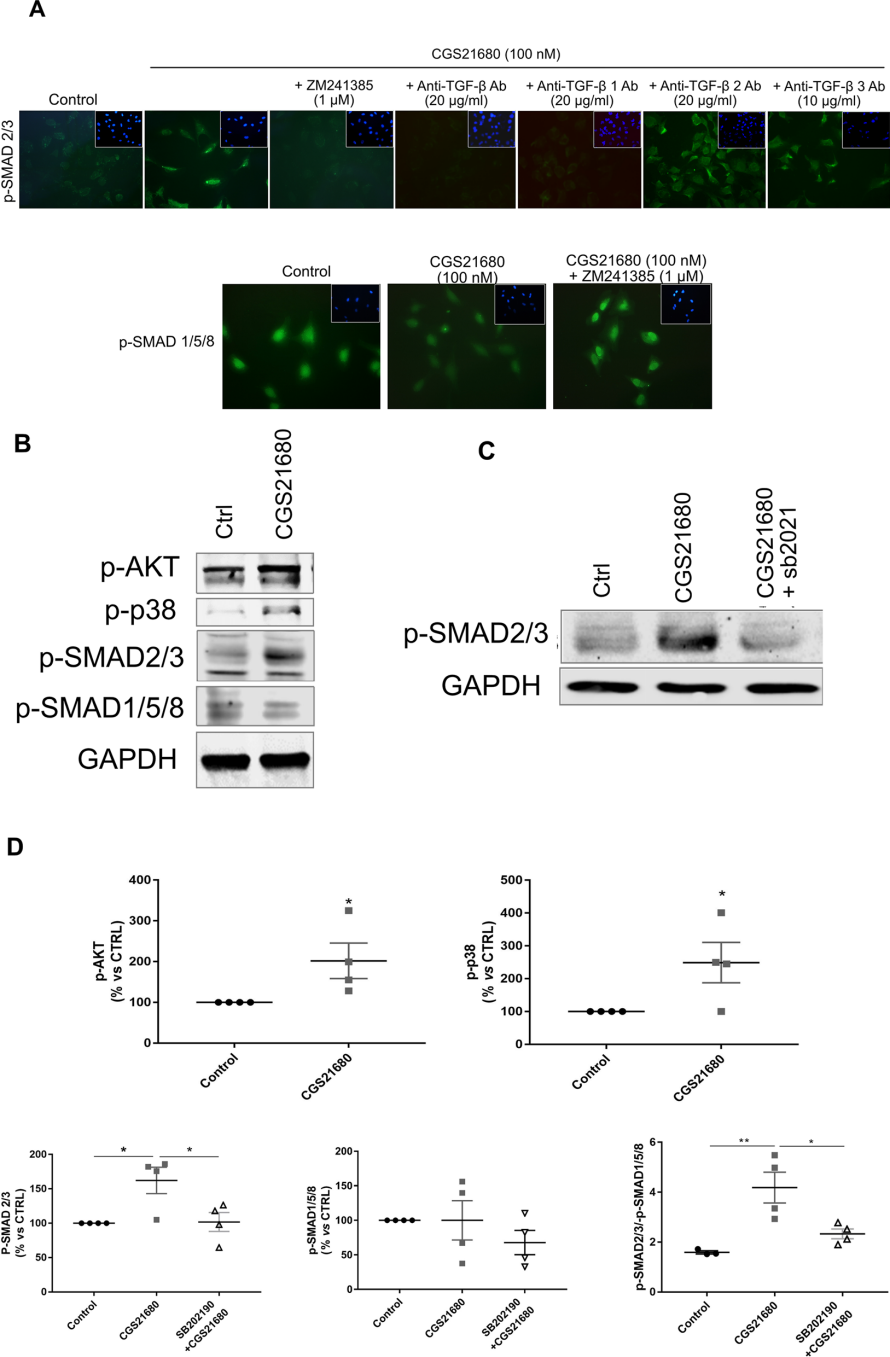
A2AR ligation stimulates P-SMAD2/3 and downregulates P-SMAD1/5/8 nuclear translocation by a mechanism involving TGF-β1 and, partially, TGF-β2 and 3. (**A**) Incubation of human chondrocytic cells with CGS21680 (100 nM, 30 min) induces P-SMAD2/3 translocation to the nucleus and reduces SMAD1/5/8 nuclear localization. Cells were pre-incubated for 15 min. with anti-TGF-β, anti-TGF-β1, anti-TGF-β2 and anti-TGF-β3 prior to incubation with CGS21680 (100 nM, 30 min) with or without ZM241385 (1 µM). (**B**) Representative images of Western Blots of phosphorylated AKT, P38, SMAD2/3, SMAD1/5/8 and GAPDH in TC28a2 chondrocytes. (**C**) SMAD2-3 phosphorylation is inhibited by pre-incubation with the p38MAPK inhibitor SB202190 (10 µM, 90 min pre-incubation). (**D**) Relative quantitation of p-AKT, p-P38MAPK, p-SMAD2/3, p-SMAD1/5/8 and the ratio of p-SMAD2/3/p-SMAD1/5/8. (n = 4 for each group; *p < 0.05; ** p  < 0.01).

### Lipo-CGS treatment increases TGFβ expression in cartilage

As noted above, genome-wide expression analysis indicated that Lipo-CGS treatment decreased expression of TGFβ2 and 3, so we next determined whether there was any change in TGFβ expression in the cartilage of the rats with established PTOA treated with Lipo-CGS. In the deep layers of cartilage, we observed an increase of TGFβ expression in the Lipo-CGS-treated group. In the uninvolved knee the expression of TGFβ was present but limited to the chondrocytes located in the more superficial region, whereas TGFβ expression was reduced in the Saline- and Lipo-treated group (Fig. 5A).

Because TGFβ is present in the matrix in many tissues and must be activated to stimulate cells, we determined whether the chondrocytes had been stimulated by active TGFβ by determining whether there was nuclear localization of phospho-SMADs, critical mediators of TGFβ signaling. We observed that phospho-SMAD1/5/8 was present in the nuclei of chondrocytes in normal, saline- and Lipo-treated knees (Fig. 5B). In contrast, we were surprised to observe that Lipo-CGS treatment stimulated a decrease in nuclear phospho-SMAD1/5/8 and increased nuclear localization of phospho-SMAD2/3. Nuclear phospho-SMAD2/3 was not observed in unaffected knees or knees treated with either saline or Lipo (Fig. 5B). In mice with obesity-induced OA, we also observed that Lipo-Ade and Lipo-CGS stimulated TGFβ expression although the change was not as marked as in the rats with established PTOA (Fig. 5C). Similarly, Lipo-CGS and Lipo-Ade treatment reduced nuclear phospho-SMAD1/5/8 expression and stimulated an increase in nuclear phospho-SMAD2/3 (Fig. 5C). These results indicate that stimulation of A2AR alters not only TGFβ expression and activation but also regulates the downstream signaling of TGFβ.

### A2AR stimulation directly affects phosphorylation of SMAD2/3 and SMAD1/5/8 via activation of AKT and p38 MAPK

The effect of intra-articular injections of Lipo-CGS and Lipo-Ade on TGFββ expression and the change in TGFβ signaling in the knees of rats with established PTOA and mice with established obesity-induced OA was surprising. Moreover, it is possible that the changes observed in TGFβ signaling in vivo might be secondary to other changes induced by the treatments. Therefore, we tested the effect of the selective A2AR agonist CGS21680 on nuclear localization of phospho-SMADs in T/C-28a2 cells, a human chondrocyte cell line. In untreated cells phospho-SMAD1/5/8 was present in the nuclei and there was no phospho-SMAD2/3 present (Fig. 6A). After incubation with CGS21680 for 30 min, there was an increased nuclear translocation of phospho-SMAD2/3 and a diminished nuclear phospho-SMAD1/5/8, as detected by immunofluorescence (Fig. 6A). The selective A2AR antagonist ZM241385 (1µM) completely reversed the effect of CGS21680 on nuclear localization of phospho-SMADs. The presence of a neutralizing pan-TGFβ antibody or a more specific TGFβ1 antibody blocked the A2AR agonist-induced P-SMAD2/3 phosphorylation. Antibodies directed against TGFβ2 and anti-TGFβ3 antibody partially reversed nuclear localization of phospho-SMAD2/3 (Fig. 6A).

Western blotting analysis also revealed a TGF-β independent pathway of activation for *p* SMAD2/3 that changes the balance of intracellular p-SMAD2-3/p-SMAD1-5-8. Previous reports indicate that activation of p-38MAPK phosphorylate and activate pSMAD2/3 by phosphorylation of AKT^17,18^ and we have previously demonstrated that A2AR stimulation leads to activation of p-38MAPK in human fibroblasts and stellate cells^19-22^. Treatment of chondrocytic cells with the selective A2AR agonist CGS21680 induced an increase of cellular p-p38 MAPK and p-AKT (Fig. 6B). Moreover, the effect of the A2AR agonist on SMAD2/3 phosphorylation and activation was reversed by pre-incubation with the inhibitor of p38 MAPK, SB202190 (Fig. 6C and D). Although we found no significant reduction in whole cell p-SMAD1/5/8, the ratio of p-SMAD2-3/p-SMAD1-5-8 increased in the control group from 1.6 to 4.2 in CGS21680 treated cells (*p* < 0.01, Fig. 6D); administration of SB202180 decreased the effect of CGS21680 on the ratio p-SMAD2-3/p-SMAD1-5-8 to 2.33 (*p* < 0.05, Fig. 6D).

## Discussion

**Figure 7:**
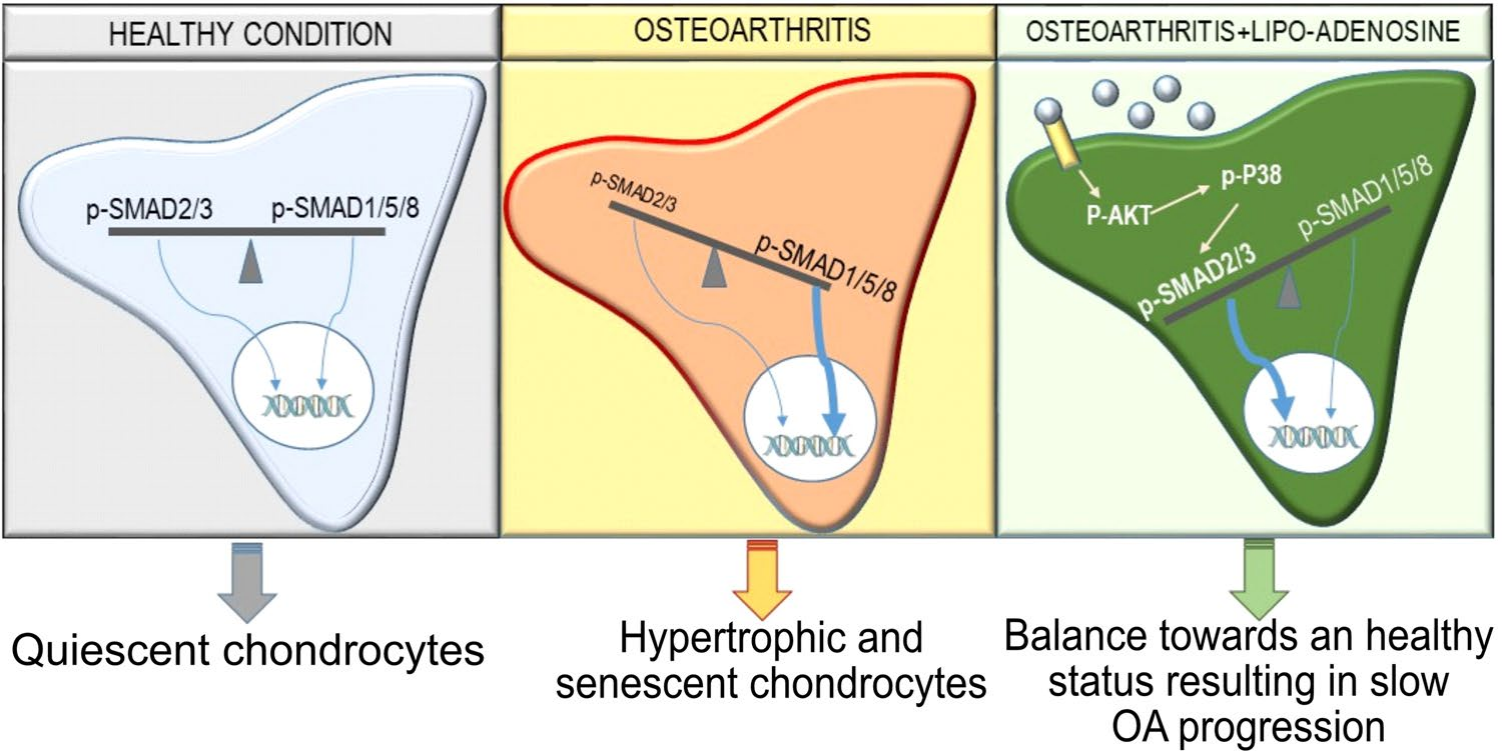
Activation of A2AR signaling increases SMAD2/3 phosphorylation through p-AKT/p-P38 dependent mechanism. Schematic representation of molecular pathway activated by adenosine (grey circle) after A2AR ligation involving phosphorylation of AKT and P38 leading to increased SMAD2/3 phosphorylation compared to SMAD1/5/8 phosphorylation inducing an imbalance in the ratio p-SMAD2-3/p-SMAD1/5/8 which leads to activation of genes involved in cell proliferation and new cartilage synthesis.

We have previously reported that adenosine and its A2AR play a central homeostatic role in cartilage and chondrocytes during periods of physiological mechanical stress and pathological inflammation^11^. We also demonstrated that replacement of adenosine prevents cartilage damage in a rat model of post-traumatic OA^11^. Here we present evidence that activation of A2AR by intra-articular injection of liposomal adenosine and the potent and specific A2AR agonist CGS21680lows OA progression in two different models of established OA, a murine model of obesity-induced OA and a rat model of PTOA. Moreover, our data suggest that A2AR stimulation alters both TGFβ activation and post-receptor signaling from a pathway associated with chondrocyte hypertrophy and terminal differentiation (ALK1/P-SMAD1/5/8) to a pathway associated with chondrocyte proliferation and matrix production (ALK5/P-SMAD2/3; Fig. 7).

Adenosine and its A2AR play a critical role in maintaining cartilage and joint homeostasis by inhibiting cartilage degradation and promoting cartilage matrix production by chondrocytes^11-14^. The source of adenosine in the extracellular space is primarily from ATP transported into the extracellular space where it is hydrolyzed to adenosine by ecto-enzymes. With age, inflammation and injury ATP levels in chondrocytes and other cell types fall, likely as a result of mitochondrial dysfunction^23^, and lower levels of ATP are transported into the extracellular space. The resulting diminution of A2AR stimulation permits chondrocyte hypertrophy and expression of proteins, like matrix metalloproteases, that contribute to development of OA. In contrast, in inflamed and injured cartilage cytokines enhance both A2AR expression and function^24-26^, thus magnifying the functional effects of A2AR stimulation. As a result, it is likely that the marked changes in chondrocyte function induced by liposomal adenosine and CGS21680 in the setting of OA are greater than expected in otherwise normal chondrocytes.

Although it is likely, based on the efficacy of the lipo-CGS21680 (a selective A2AR agonist) treatments to promote cartilage formation, that stimulation of the A2AR is principally responsible for the improvement in osteoarthritis observed here it is possible that other adenosine receptors are also involved in this therapeutic effect. Deletion of both A2AR and A3R have been shown to lead to osteoarthritis^11,27^ and it is likely that the homeostatic role of adenosine in cartilage and chondrocytes is mediated by both receptors. Interestingly osteoarthritis becomes evident within 6 months of age in A2AR deficient mice but has only been described in aged mice lacking A3R, suggesting that the A2AR is more critical for maintenance of chondrocyte homeostasis throughout life, whereas loss of the A3R exacerbates the effects of age on cartilage. Agents that stimulate A2AR and A3R prevent progression of osteoarthritis in animal models of osteoarthritis induced by trauma and injection of monoidoacetate, respectively. The anti-inflammatory properties of stimulating both adenosine receptors ^28^ likely play a critical role in preventing progression of osteoarthritis in these models, particularly the monoiodoacetate-induced model of osteoarthritis which is characterized by acute inflammatory changes. Both A2AR and A3R stimulation inhibit chondrocyte production of catabolic proteins^11,14,27,29,30^ associated with cartilage destruction but, to date, only A2AR stimulation of chondrocytes induces production of cartilage matrix, as shown here.

The results presented here are consistent with the hypothesis that A2AR stimulation reverses OA, in part, by stimulating TGFβ production in vivo and shifting downstream signaling to promote matrix production. We found, in both in vitro and in vivo studies, that stimulation of A2AR shifts TGFβ signaling to a pathway that blocks chondrocyte hypertrophy and promotes cartilage repair. When studied in vivo*,* we observed that there was an increase in TGFβ immunostaining in the chondrocytes of rats with PTOA treated with liposomal adenosine and CGS21680, although expression analysis showed diminished message for TGFβ in chondrocytes harvested from these same animals. Nonetheless, in in vitro studies using a chondrocyte cell line, we observed that A2AR stimulation increased nuclear localization of p-SMAD2/3 by a mechanism that was blocked by antibodies to TGFβ1 and, to a lesser extent, TGFβ2/3 within a timeframe during which it is most likely that the principal effect of the A2AR stimulation is on activation of TGFβ rather than increased expression. More interestingly, A2AR stimulation biases TGFβ signaling via activation of p38 MAPK, a signaling intermediate that is activated by A2AR stimulation and downstream AKT activation^20,21,31^.

In previous studies we have observed that A2AR signal, in part, via AKT and p38MAPK^31^ in association with activation of Wnt signaling in fibroblasts^22,32^. Wnt signaling in chondrocytes is associated with terminal differentiation of chondrocytes and catabolism of cartilage^33,34^. We found here that in chondrocytic cells A2AR stimulation also leads to activation of AKT but the consequences of this activation include a shift in TGFβ signaling from a catabolic pathway (SMAD1/5/8) to an anabolic pathway (SMAD2/3) which, in these studies, appears to be dominant over the Wnt-mediated catabolic pathway observed previously. Likely other cAMP-dependent signals contribute to this effect as well although these have not yet been explored.

Prior studies have demonstrated that activation of latent TGFβ is crucial for maintaining chondrocyte and cartilage homeostasis^35^. TGFβ is released into the extracellular space in a latent form and, once activated, TGFβ regulates cellular function by binding the ALK5 receptor leading to phosphorylation of SMAD2/3 and the stabilization of the transcription factor SOX9 and reduction of RUNX2 activity^36,37^. ALK5 activation and P-SMAD2/3 exert a positive effect on cartilage by regulating chondrocyte proliferation and blocking chondrocyte hypertrophy^38-40^. Accordingly, with aging and joint inflammation there is a decrease in SMAD2/3 phosphorylation^41,42^. Chondrocytes in adult animals also express ALK1, which is activated by TGFβ and other TGFβ-related ligands leading to phosphorylation and nuclear localization of SMAD1/5/8^43^. In contrast to ALK5 stimulation, ALK1 activation is associated with an increase in hypertrophic chondrocytes^44^. Thus, TGFβ stimulation plays different roles during cartilage development and maturation and later during aging and senescence.

Although a number of agents have been reported to prevent OA progression in animal models, there are very few examples of agents that reverse OA changes in cartilage. Intermittent activation of PTH signaling has been reported to repair articular cartilage damage^45^. Similarly inhibition of Wnt signaling is also reported to prevent cartilage degradation and promote cartilage regeneration^46^, and enhancing the levels of the nuclear transcription factor FoxO1 promotes regeneration in OA cartilage^47^. Here we observed that intraarticular injection of either liposomal preparations of adenosine or a selective A2AR agonist promotes cartilage repair and regeneration in the setting of established OA in both rats with PTOA and mice with obesity-induced OA. It is unlikely that A2AR stimulation leads to inhibition of Wnt signaling in chondrocytes since we have previously shown in primary human dermal fibroblasts and in murine models of fibrosis that A2AR stimulation promotes Wnt signaling^19,48^.

In the obesity-induced model of osteoarthritis in mice the pro-inflammatory cytokines adiponectin, resistin and IL-6^49-52^ contribute to the chondrocyte and cartilage defects observed and these and other adipokines have been proposed as biomarkers of osteoarthritis. Despite the contribution of these cytokines to the pathogenesis of OA, A2AR stimulation reverses the osteoarthritis that develops in obese mice without affecting serum levels of these adipokines. Stimulating A2AR diminishes responses to a number of inflammatory cytokines and reduces the production of inflammatory cytokines^28^ and our results indicate that intraarticular treatment with either Lipo-Ade or Lipo-CGS diminishes the responses of chondrocytes and other synovial cells to these inflammatory stimuli.

Currently there are no agents approved for the promotion of cartilage regeneration in OA patients. Our results, though preliminary and in need of further and deeper investigation involving other OA animal models, indicate that the adenosine A2AR is a novel target for a disease-modifying OA drug.

## Methods

### Materials

ZM241385 (A2AR antagonist) and CGS21680 (A2AR agonist) were obtained from TOCRIS (MI, USA). Antibodies: anti-TGFβ (3711S), anti p-AKT (4060), p-P38 (4511S) were purchased from Cell Signaling (MA, USA); ADAMTS5, phospho-SMAD1/5/8 antibody (AB3848), multiplex kit for mouse adipokines detection (MADKMAG-71 K) from Millipore (MA, USA);anti-Rb-Col2A1 (sc-28887), and secondary antibody HRP conjugate (SC-2030) were obtained from SantaCruz (CA, USA). Goat Anti-Type I Collagen (1310-01) was purchased from Southern Biotech (Birmingham, AL). SB202190 was purchased from R&D System (Minneapolis, MN). Hexabrix was purchased from Guerbet (IN, USA); anti-p-SMAD2-3 was purchased from Abcam (Cambridge, UK). Paraformaldehyde (PFA) 32% was obtained from Electron Microscopy Sciences (PA, USA). Ethylenediaminetetraacetic acid (EDTA), bovine serum albumin, SIGMA*FAST* 3,3′-Diaminobenzidine tablets, Anti-Rabbit IgG–FITC antibody, cholesterol, phosphatydil choline from egg yolk, ethanol, glycerol, adenosine and ascorbic acid, DAPI, primers for RT-PCR (sequences in Table 2) were purchased from Sigma Aldrich (MO, USA). DMEM, penicillin–streptomycin and fetal bovine serum were purchased from Life technology (NY, USA). The kit for RNA extraction was purchased from QIAGEN (CA, USA). The reverse transcription kit was purchased from Applied Biosystem (CA, USA). The SYBR Green PCR Master Mix was obtained from AB Applied Biosystems (CA, USA). Anti TGFβ 2 and 3 were purchased from R&D Systems (Minneapolis, MN).

**Table 2:** Primer sequences for gene expression analysis using qRT-PCR.

*Adamts14*	Forward	5′-aagctggaactcagccgtta-3’	*SOX9*	Forward	5′-ctagccttccttgcaaccag-3’
	Reverse	5′-aaggttctgaaagccaagca-3’		Reverse	5′-aatctgagagcctggggaat-3’
*AHRR*	Forward	5′-cagcaacatggcttctttca-3’	*Tgfb2*	Forward	5′-aatcctagccagggacgttt-3’
	Reverse	5′-gaagcactgcattccagaca-3’		Reverse	5′-tgcaggagcaaaaaggttct-3’
*Btg2*	Forward	5′-agctgtgaatcgctccagat-3’	*Tgfb3*	Forward	5′-gcaacttggaggagaactgc-3’
	Reverse	5′-agacaggcctgctcaacag-3’		Reverse	5′-gtcagaggctccaggtcttg-3’
*Cnmd*	Forward	5′-aagcagtgctccctctacca-3’	*Acan*	Forward	5′-ctacgacgccatctgctaca-3’
	Reverse	5′-ctctctccttcctgctggtg-3’		Reverse	5′-gctttgcagtgaggatcaca-3’
*Col1a1*	Forward	5′-tgctgccttttctgttcctt-3’	*Gapdh*	Forward	5′-agacagccgcatcttcttgt-3’
	Reverse	5′-aaggtgctgggtagggaagt-3’		Reverse	5′-cttgccgtgggtagagtcat-3’
*Col3a1*	Forward	5′-gtccacgaggtgacaaaggt-3’			
	Reverse	5′-catcttttccaggaggtcca-3’			

### Animals

Mice and rats employed in this study were kept under regular lighting conditions (12 h light/dark cycles) and given food and water ad libitum. Sprague Dawly male rats were used in experimental OA induction (n = 5–6 for each group). Twelve week old, male C57/Bl6 mice were used in the obesity-induced OA model. Newborn mice, 5 days old were used for chondrocyte isolation from femoral and knee joint.

### Murine model of obesity related OA

C57/Bl6 mice, 12 weeks old, were fed a high fat diet (60% fat, Research Diets D12492i—Research Diets Inc., NJ-USA) for 12 weeks before and during intraarticular treatment with saline or liposome with and without CGS21680 or adenosine. Injections (10 µl) were administered intraarticularly every 10 days for 6 injections in total. This intraarticular injection regimen was chosen arbitrarily based on the assumption that OA progression would be ongoing and that the effects of the liposomal injections would be finite. Animals were monitored with DEXA scan (Lunar PIXImus, GE Lunar Corp., WI-USA) on a monthly basis to assess differences in percent total body fat and bone mineral composition between groups. Legs and plasma were collected at the end-point.

### PTOA induction in rats and liposomal adenosine treatment

The post-traumatic OA (PTOA) model used is a non-invasive method for inducing anterior cruciate ligament (ACL) rupture in rat knees in vivo with a single load of tibial compression. The procedure was performed under anesthesia (1–3% isoflurane) as previously described^15^. All experimental groups of rats, as described below, consisted of 3 rats and each experimental group was repeated once (total of 5–6 rats per experimental group). This number of rats was chosen because larger group sizes led to operator overload and diminished quality of results. To have a > 90% power to detect a 70% reduction in OARSI score using an ANOVA with repeated measures, with an α error probability of 0.05 and three groups, we will need a minimum of six animals for each condition. OARSI score variance for previously published experiments was used for the power analysis^11^.

Starting 4 weeks after ACL rupture when osteoarthritis is clearly present^15^, rats were treated every 10 days with intra-articular injections of 100 µl of a liposomal suspension containing CGS21680 or adenosine (1 mg/Kg), empty liposomes or with saline for 8 weeks. This intraarticular injection regimen was chosen arbitrarily based on the assumption that OA progression would be ongoing and that the effects of the liposomal injections would be finite. Knee swelling and weight in the rats were measured before every injection. At the end of the experiment rats were sacrificed and both legs were harvested for immunohistochemistry and micro-computed tomography (µCT) analysis.

### Liposome preparation and measurement of adenosine encapsulation

Liposomes were prepared fresh the day before injection. Ethanol was added to soybean oil containing adenosine or CGS21680. The lipid phase containing phosphatidyl choline and cholesterol (1:0.5 by molar ratio) was added to the previous solution and emulsified at 15,000 rpm for 10 min. Saline along with glycerin was then added to the lipid phase and was homogenized at 15,000 rpm for 20 min followed by sonication for 1 min at 100% duty cycle. Adenosine encapsulation in liposome was measured after 1, 2, 24, 48 h incubation at 37 °C (Supplemental Fig. 1). Liposomes were incubated in PBS and then collected at the different time points. Liposomes were centrifuged at 23000 g for 1 h at 4 °C. Supernatant was removed and the liposome pellet was resuspended in a saline solution containing 0.5% Triton-X100. Adenosine concentration in the remaining intact liposomes was quantified by High Performance Liquid Chromatography.

### Luminex assay

Mouse plasma samples were assessed for 6 analytes (IL-6, Insulin, Leptin, MCP-1, PAI-1, Resistin, TNF-α) by using multiplexed bioassays and the Luminex xMAP technology following the manufacturer’s instructions. The immunoassay was read by using MAGPIX analyzer in the Translational Research Laboratory Instrument Core at NYU-Langone Health.

### Histology, immunohistochemistry and immunofluorescence

Both limbs were cleaned of soft tissue, placed into 4% PFA for 48 h then preserved in 70% ethanol. After µCT analysis the samples were washed with PBS and decalcified in 10% EDTA for 4 weeks. Mouse samples were fixed and decalcified for 3 weeks. Paraffin-embedded histological coronal Sections (5 µm) were cut, mounted and prepared for analysis with H&E and Safranin O/Fast green staining to assay different cartilage components (3 slices, 200 um apart were analyzed for each animal). TGFβ, ADAMTS5, collagen-1 and collagen-2 were detected in cartilage by immunohistochemistry. Briefly, joint sections were deparaffinized by xylene and re-hydrated in decreasing ethanol concentrations. Antigen retrieval was performed by immerging the sections in a proteinase K solution (20 µg/ml) for 30 min at 37 °C. Sections were depleted of endogenous peroxidase activity with 3% H_2_O_2_ in methanol, then blocked with PBST containing bovine serum albumin (1%) and FBS (5%) for 60 min. Sections were incubated overnight with rabbit antibody (1:200 dilution) specific for each protein under study. After rinsing with phosphate buffered saline (PBS), horseradish peroxidase (HRP)-conjugated secondary antibody was applied and stained with diaminobenzidine (DAB) kit. Slides were scanned using a Leica microscope equipped with Slidepath Digital Image Hub version 3.0 Software (www.leicabiosystems.com). Assessment of OA was performed by evaluation of Safranin-O stained slides in a blinded fashion. OARSI score was determined blindly as previously described^53^. Briefly, for histologic scoring, slides were stained using Safranin-O Fast Green technique. OARSI score determined blindly for each specimen taking into account the severity of cartilage degradation, cartilage calcification, presence of osteophytes and their size^54^.

Immunofluorescence was performed in order to detect P-SMAD2/3 and P-SMAD1/5/8. After over-night incubation with the primary antibody, slides were incubated with the FITCH secondary antibody (1:200) for 1 h. Slides were mounted with mounting media containing DAPI for nuclei staining. Tissue slide preparation and H&E staining were performed in the Experimental Pathology Research Laboratory at NYU-Langone Health.

### µCT Cartilage examination

After washing with PBS, rat knees (femoral and tibial surfaces from joints previously dislocated, n = 5–6 for each group) were incubated in PBS containing the ionic contrast agent Hexabrix (40% v/v) for 6 h. All joints were evaluated in a (16 mm) scanning tube providing a volex size of 10.5 µm and scanned at 55 kV, 181 µA, and 110 min of acquisition time^11^. During scanning the samples were wrapped in paper soaked in PBS to avoid dehydration. uCT scanning and data reconstruction were performed by the microCT core at NYU-School of Dentistry.

### P-SMAD2/3 and P-SMAD1/5/8 detection in human chondrocyte cell line

Human immortalized chondrocyte cells (T/C-28a2) were a kind gift of Dr. Mary B. Goldring, from Hospital for Special Surgery, NY-USA. Cells were cultured in complete medium DMEM-F12 containing 10% FBS and 1% Penicillin–Streptomycin. Cells were grown at 37 °C, in 5% CO2. T/C-28a2 cells were treated with CGS21680 for 30 min with a pre-incubation of IL-1β (10 ng/ml for 60 min) and specific TGFβ neutralizing antibodies (20 ug/ml for pan anti-TGFβ, -TGFβ 1 and 2; 10 ug/ml for anti-TGFβ 3).

Cells were plated in 8-well chamber slides and, after the appropriate treatments, were washed with cold PBS and fixed with paraformaldehyde 4% (10 min). Cells were permeabilized using a solution of PBS containing Triton 0.25% for 10 min. After 3 washes for 5 min each, a blocking solution (FBS 5%, BSA 1% in PBST) was added to the cells for 1 h. Cells were incubated with primary antibody against P-SMAD2/3 and P-SMAD1/5/8. Cells were washed 3 times for 5 min each with PBS and incubated with the secondary antibody FITC conjugate (1:200 in PBST) for 1 h. After 3 washes of 5 min each, a cover slide was applied to the slide with a mounting media containing DAPI. Immunofluorescence was revealed by the Nikon Eclipse Ni fluorescence compound microscope^11^.

For western blotting analysis, cells were pre-incubated with p38 MAPK inhibitor SB202190 (10 µM for 90 min) and then treated for 30 min with CGS21680 (100 nM).

After cell treatments, the total protein extracts were collected and stored at − 80 °C. Total protein fractions were quantified using the DC Protein assay kit (BioRad). Western blotting was performed by electrophoresing 30 µg protein through a 10% polyacrylamide gel followed by transfer of proteins to nitrocellulose membranes. Nitrocellulose membranes were incubated overnight at 4 C with the specific primary antibody (1:1000) and, after washing, incubated with goat anti-rabbit IRDye 800 CW and goat anti-mouse IRDye 680 RD (1:5000). Membranes were scanned with Li-Cor Odyssey equipment and the intensities of the protein bands were quantified by densitometric analysis using Image Studio software version 2.0.38 (www.licor.com)^55^.

### A2AR stimulation in murine chondrocytes

Chondrocytes were isolated from articular cartilage as previously described^11^. Cells were treated with CGS21680 (1 µM) alone or in the presence of the A2AR antagonist ZM241385 (1 µM) for 7 days in order to assess aggrecan production by using Alcian Blue staining. The Alcian Blue staining was performed how previously described^56^.

Cells were isolated for RNA and Real Time PCR after 24 h of treatment with IL-1β (5 ng/ml) alone or in presence of CSG21680 (1 µM).

### Reverse transcription and real time PCR

RNA extraction was performed from mouse primary chondrocytes using RNeasy Mini Kit (Qiagen, Invitrogen) and QIAshredder colums (Qiagen, Invitrogen), following the manufacturer’s protocol. RNA reverse transcription was performed using the MuLV Reverse Transcriptase PCR Kit (Applied Biosystems). After RNA reverse transcription to cDNA, real time PCR reactions were performed^11^.

### Genome wide expression analysis of chondrocytes isolated from OA rats after Lipo-CGS treatment

Rats were treated with saline, Lipo and Lipo-CGS following the timing previously described. Animals were sacrificed at the end of the experiment and knee articular chondrocytes were collected. Briefly, cartilage was sliced using a scalpel and incubated with pronase 1% for 90 min, then washed and incubated with 0.4% in collagenase for 3 h at 37C. Each sample was obtained by combining tissues from 3 rats. Cell suspension was washed with PBS and cultured. RNA extraction was performed using the RNA extraction kit (QIAGEN) following the manufacturer instruction.

The Illumina HiSeq 4000 was used to generate single-read 50 bp RNA-Seq data. FASTQ files were generated using the bcl2fastq2 Conversion software (version 2.17; www.support.illumina.com) to convert per-cycle BCL base call files. The alignment program, STAR (version 2.4.5a; https://github.com/alexdobin/STAR/releases)^57^, was used for mapping reads to the *Rattus norvegicus* reference genome, Rnor_6.0 and FastQ Screen (version 0.5.2; https://www.bioinformatics.babraham.ac.uk/projects/fastq_screen) was utilized to check for contaminants. The software, featureCounts (Subread package version 1.4.6-p3; https://subread.sourceforge.net/), was used to generate the matrix of read counts for annotated genomic features. For differential expression analysis and the generation of normalized expression values, the DESeq2^58^ package (Bioconductor version 3.3; https://bioconductor.org/news/bioc_3_3_release/) was utilized in the R statistical programming environment. DESeq tests for differential expression by fitting a negative binomial distribution to gene counts which adequately captures the spread of biological replicates. Further data transformations, plotting, and statistical filtering were also performed in the same R environment from custom scripts. *p* Values attained from differential gene expression analysis were adjusted for multiple testing by controlling for false discovery using Benjamini-Hochberg’s method^59^. Genes with adjusted *p* values < 0.05 and a fold-change of 1.5 were flagged as differentially expressed and then used for Gene Set Enrichment Analysis (GSEA)^60,61^. GSEA was performed using the latest DAVID Bioinformatics Resource (version 6.8; https://david.ncifcrf.gov/)^62^ to elucidate over-represented biological processes and pathways.

### Data analysis

Amira software version 6.3 (Visage Imaging GmbH, Berlin, Germany; https://www.fei.com/software/) was used to reconstruct rat joints from µCT data based on differential density of bone and Hexabrix-treated cartilage. Statistical significance for differences between groups was determined using Student’s T-test, two-way or one-way ANOVA, as appropriate, using GraphPad Prism software version 7 (GraphPad, San Diego, CA; https://www.graphpad.com/). If the overall differences were significant (F < 0.05) then differences between groups were analyzed by Bonferroni post-hoc testing.

### Study approval

All protocols for experimental procedures involving the use of animals were approved by the New York University School of Medicine Institutional Animal Care and Use Committee. All methods were performed in accordance with New York University Medical Center guidelines and regulations.

## Supplementary information

Supplementary file1.

## Data Availability

The data that support the findings of this study are available from the corresponding author upon request. Original data from genomic analyses are posted on https://github.com/FenyoLab/Cronstein.
