# Supplementary material for: Intraarticular injection of liposomal adenosine reduces cartilage damage in established murine and rat models of osteoarthritis
Source: Sci Rep. 2020 Aug 10;10:13477. doi: 10.1038/s41598-020-68302-w (PMC7418027; doi:10.1038/s41598-020-68302-w)

Intraarticular injection of liposomal adenosine reduces cartilage damage in established murine and rat models of osteoarthritis

[1]Carmen Corciulo, [1]Cristina Castro, [2]Thomas Coughlin, [3,4]Samson Jacob, [3,4]David Fenyö, [5]Daniel B. Rifkin ,[2,6]Oran D. Kennedy, [1,7]Bruce N. Cronstein

[1]Department of Medicine-Division of Translational Medicine-NYU School of Medicine, 550 First Avenue, New York, New York 10016, USA

[2]Department of Orthopedic Surgery-NYU School of Medicine, 550 First Avenue, New York, New York 10016, USA

[3]Institute for Systems Genetics-NYU School of Medicine- 435 East 30th Street, New York, New York 10016, USA

[4]Department of Biochemistry and Molecular Pharmacology-NYU School of Medicine- 435 East 30th Street, New York, New York 10016, USA

[5]Department of Cell Biology- NYU School of Medicine, 550 First Avenue, New York, New York 10016, USA

[6]Department of Anatomy- The Royal College of Surgeons in Ireland, 123 St Stephens Green, Dublin 2, Ireland

[7]Department of Medicine-Division of Rheumatology-NYU School of Medicine, 550 First Avenue, New York, New York 10016, USA

Corresponding author:
Bruce Neil Cronstein
NYU Langone Health
Medical Science Building, 550 1st Avenue, New York, NY 10016
Telephone number: +1 (212) 263-6404
e-mail address: bruce.cronstein@nyumc.org

Supplemental figures

Supplemental figure 1

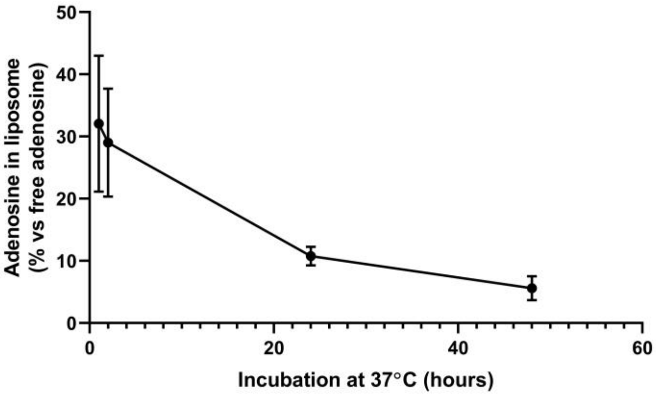

S1- **Adenosine remaining in liposomes after incubation at 37°C.** The graph shows percentage of adenosine encapsulated in liposomes after 1, 2, 24 and 48 hours of incubation in PBS at 37ºC (Data are expressed as mean±s.e.m; n=4 for each time point).

Supplemental figure 2

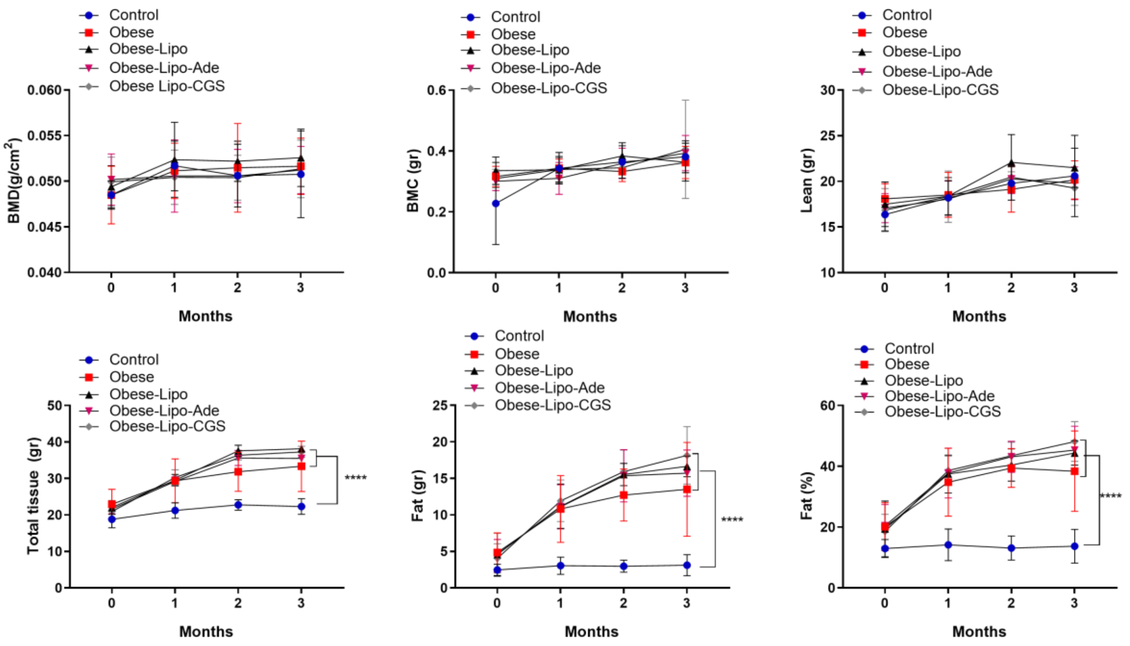

S2- **Body composition of obese mice.** DEXA scan analysis was performed in obese mice treated with empty-liposomes, Lipo-Ade and Lipo-CGS at different time point from the beginning of the high fat diet until the end of the experiment. As expected fat tissue and total tissue were significantly higher in obese mice compare to those fed a control diet. Bone composition did not change with the diet. No differences in weight gain or % body fat were detectable between the various treatment groups (n=5 for each group).

Supplemental figure 3

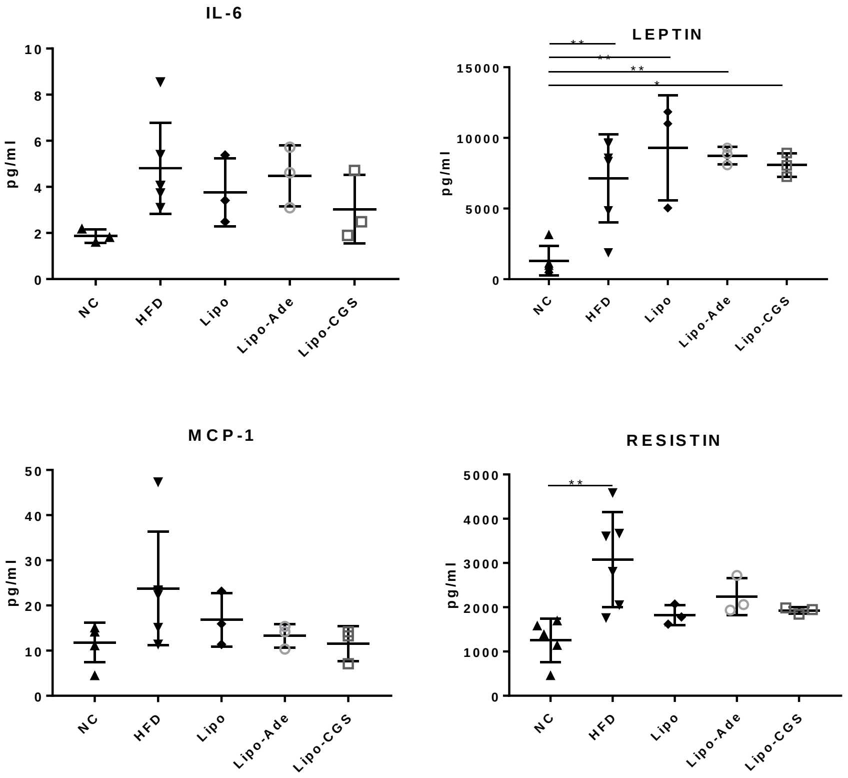

S3- **Systemic inflammation in obese mice.** Increased levels of IL-6, Leptin and Resistin were detected in the plasma of mice with obesity-induced osteoarthritis. The local injection of liposomes did not change the systemic level of inflammation. (Data are expressed as mean±s.d. of n=3-5 for each group; *P<0.05; **P<0.01).

Supplemental figure 4

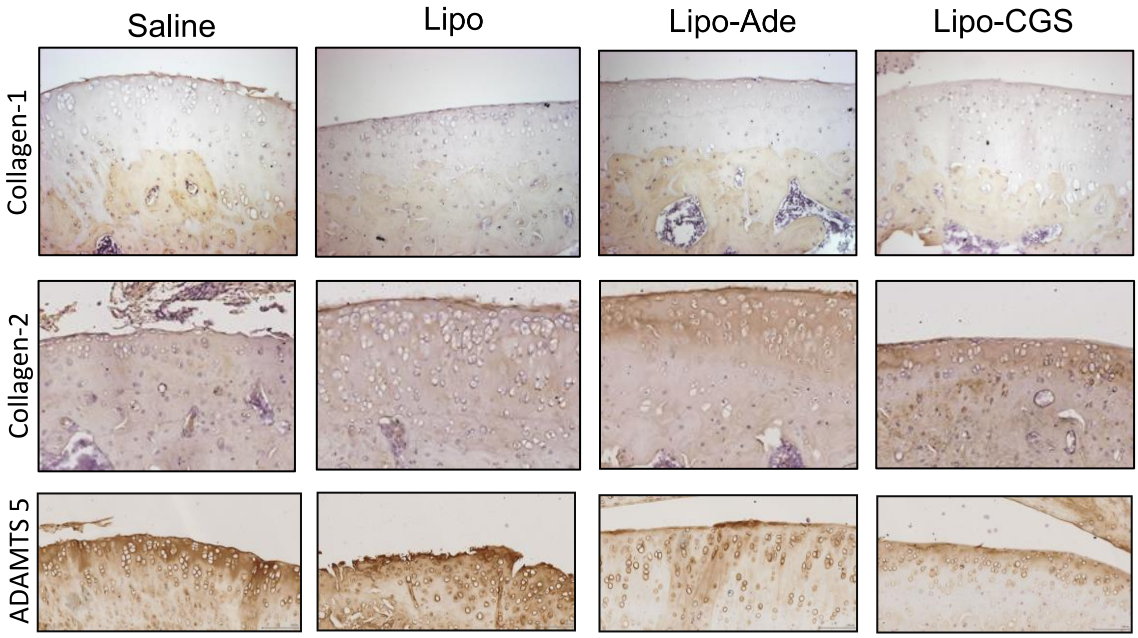

S4-**Restoring collagen 2 staining in PTOA cartilage.** Representative pictures of PTOA rat's cartilage staining for collagen-1 didn't show any differences between different groups. Knee cartilage of saline and empty-liposomes treated rats lacks Collagen-2 in the cartilage matrix. Collagen-2 in cartilage matrix increases in Lipo-Ade and in Lipo-CGS groups. ADAMTS 5 decreases in Lipo-Adenosine and Lipo-CGS21680 treated rats.

Supplemental figure 5

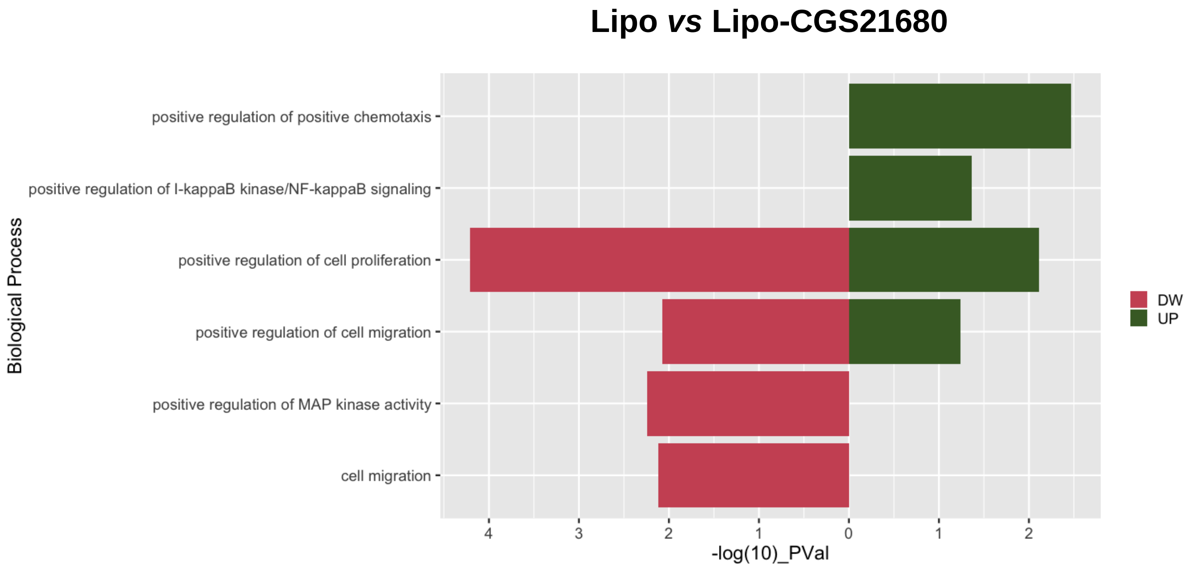

S5-**Pathway analysis of RNAseq data.** Bars in green are those obtained for chondrocytes from knees treated with empty liposomes whereas the red bars represent the changes observed in knees treated with liposomal CGS21689 injections. The pathway analysis of genes regulated by Lipo-CGS intra-articular injection suggests alteration in genes involved in cell proliferation, inflammation and MAP-kinases activity.

Supplemental figure 6

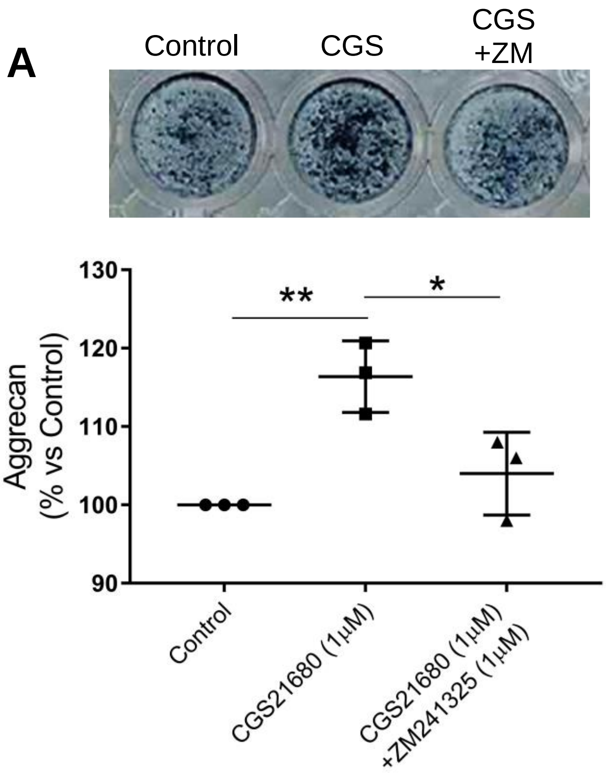

B

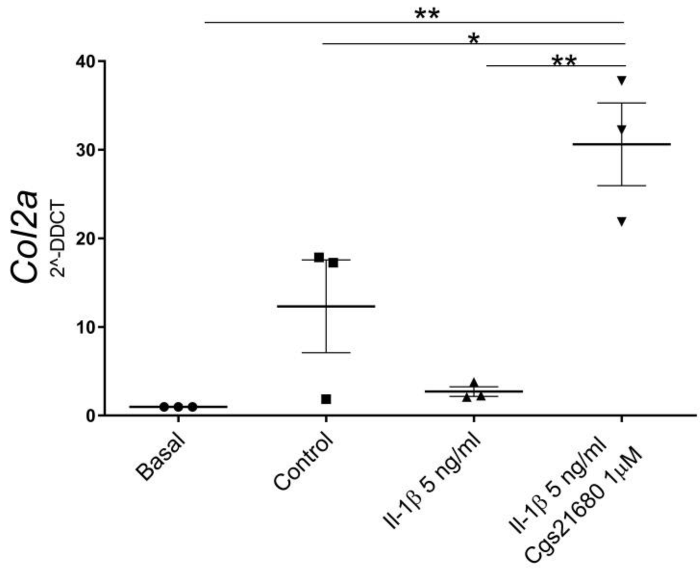

S6-**A2AR stimulation promotes aggrecan and collagen-2 synthesis in murine chondrocytes.** (A) Representative image of aggrecan staining (Alcian blue) in cell culture of primary murine chondrocytes after stimulation of CGS21680 (1 µM) and co-incubation with the A2AR antagonist ZM241385 (1 µM). Quantification of the staining is shown in the graph below. (B) A2AR stimulation prevents IL-1β mediated Col2a mRNA reduction (*, $p<0.05$; **, $p<0.01$; n=3).

Supplemental figure 7

Full inedited figure 6B-C

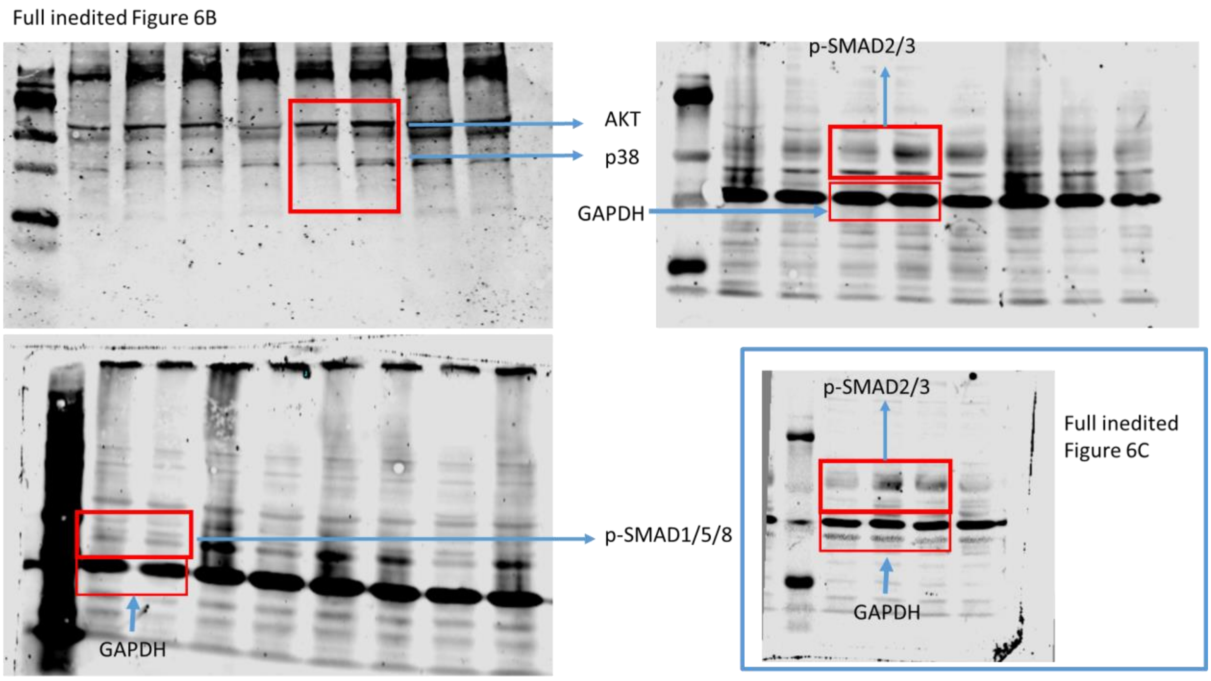

Supplement: Supplementary file 1 — Supplementary file1. [file 41598_2020_68302_MOESM1_ESM.pdf]
